# Spectroscopic and In Vitro Investigations of Boron(III) Complex with *Meso*-4-Methoxycarbonylpropylsubstituted Dipyrromethene for Fluorescence Bioimaging Applications

**DOI:** 10.3390/molecules25194541

**Published:** 2020-10-03

**Authors:** Galina Guseva, Elena Antina, Mikhail Berezin, Svetlana Lisovskaya, Roman Pavelyev, Airat Kayumov, Olga Lodochnikova, Daut Islamov, Konstantin Usachev, Sergei Boichuk, Liliya Nikitina

**Affiliations:** 1 G.A. Krestov Institute of Solution Chemistry of Russian Academy of Sciences, 1 Akademicheskaya st., 153045 Ivanovo, Russia; eva@isc-ras.ru (E.A.); mbb@isc-ras.ru (M.B.); 2 Kazan State Medical University, 49 Butlerova st., 420012 Kazan, Russia; s_lisovskaya@mail.ru (S.L.); boichuksergei@mail.ru (S.B.); nikitl@mail.ru (L.N.); 3 Scientific Research Institute of Epidemiology and Microbiology, 67 Bolshaya Krasnaya st., 420015 Kazan, Russia; 4 Kazan Federal University, 18 Kremlyovskaya st., 420008 Kazan, Russia; rpavelyev@gmail.com (R.P.); kairatr@yandex.ru (A.K.); lod_olga@mail.ru (O.L.); daut1989@mail.ru (D.I.); k.usachev@kpfu.ru (K.U.); 5 Arbuzov Institute of Organic and Physical Chemistry, FRC Kazan Scientific Center, Russian Academy of Sciences, 8 Arbuzov, 420088 Kazan, Russia

**Keywords:** dipyrromethene, BODIPY, fluorophore, biomarker, pathogenic microorganisms

## Abstract

This study focuses on the behavior of a new fluorescent marker for labeling individual biomolecules and staining cell organelles developed on a *meso*-substituted BODIPY platform. Boron(III) complex with *meso*-4-methoxycarbonylpropylsubstituted 3,3’,5,5’-tetramethyl-2,2′-dipyrromethene has been synthesized and identified via visible, UV-, NMR- and MS-spectra *X*-ray. The behavior of fluorophore in solutions has been studied with various experimental techniques. It has been found that luminophore exhibits a high quantum yield (almost ~100–75%) in the blue-green region (513–520 nm) and has high photostability. In addition, biological analysis indicates that the fluorophore exhibits a tendency to effectively penetrate into cell membranes. On the other hand, the proposed BODIPY can be used to study the significant differences among a large number of pathogens of mycotic infections, as well as to visualize structural changes in the plasma membrane, which is necessary for the clearance of mammalian cells undergoing apoptotic cell death.

## 1. Introduction

Nowadays, microscopic fungi are considered to be causing a significant increase in serious diseases in humans, animals and plants. At the same time, the number of mycoses caused by micromycetes increases annually by 5% to 10% and doubles every 10 years. In fact, human mycoses are often fatal, moreover, fungi belonging to various taxonomic groups and belonging to both pathogenic and conditionally pathogenic microorganisms act as agents of mycotic infections. These are pathogens of both humans and plants: Candida albicans, Fusarium oxysporum, Aspergillus fumigatus and many others.

In addition, microscopic fungi that can cause disease in humans are characterized by high morphogenetic flexibility and increased virulence. Hyphae formation allows them to invade and penetrate the tissues while the formation of blastoconidia helps to effectively colonize the surfaces and loci of the macroorganism. As a rule, membrane structures are involved in the process of morphological cell variability [1]. It is common knowledge that highly sensitive biocompatible fluorescent markers are crucial when it comes to visualizing the mechanisms of regulation and spread of pathogenic microorganisms and their localization sites.

For many years, chemists and biochemists have seen borofluoride complexes family of dipyrromethenes (BODIPY) as potential fluorescent markers among the well-known phosphors. However, the sufficiently hydrophobic nature of BODIPY phosphors allow them to easily penetrate through the lipid layers of cell membranes and to bind with hydrophobic fragments of proteins. Therefore, at present, studies on the use of BODIPY dyes for labeling of individual biomolecules and staining of cellular organelles’ cores are particularly actively developing [2,3,4,5,6]. 

Observing the phosphor lipophore and hydrophilicity ratio is considered a crucial step when developing new biomarkers in vivo. However, scientists in the chemistry of dipyrromethenes have always seen the wide potential of modifying the indacene backbone as an important factor for determining their interest since it allows to attach different functional substituents that can radically change the spectral characteristics of the phosphor and its lipo- and hydro-philicity for specific practical tasks. In fact, introducing substituents containing heteroatoms of various nature into the *meso*-position of the dipyrromethene domain is considered as one of the effective ways for modifying the BODIPY structures [7,8,9]. In Reference [10], the authors have shown that functionalizing BODIPY structure of dyes by introducing the dipyrromethene ligand with the ether group with an extended alkyl moiety into the *meso*-position -(CH_2_)_n_COO-CH_3_ facilitates the transport of the phosphor through the bilayer of the cell membrane and ensures the conjugation of phosphors with biologically active molecules. However, as the analysis of literature data shows [11], the disadvantage of BODIPY luminophores with an extended substituent in the *meso*-position is the uncertainty of their position in cell membranes. Such luminophores, as a rule, have two populations: a *meso*-substituent submerged for the entire length and a *meso*-substituent floating up to the membrane surface, while the extended linker bends. In many studies, this duality is unacceptable. In addition, phosphors containing nitrogen or oxygen heteroatoms are very sensitive to the properties of the medium and in biological media, are characterized by a low quantum yield of fluorescence [12,13]. The insensitivity to the polarity of solvents is very important for bioanalyzing, for the dye labeled on a biomolecule should emit stable fluorescence and be independent of various external environments. Therefore, an actual task is to search for new BODIPY biomarkers with a high quantum yield, low sensitivity of fluorescence to the properties of biological media and efficient transport across the cell membrane due to the unimpeded immersion of the molecule into the lipid bilayer. The development of such a group of phosphors will allow us to identify new structures necessary for establishing a mechanism for regulating the molecular functioning of cells and a detailed study of cell morphology. This will provide new insights necessary for understanding the physiological adaptation of multicellular organisms and will also contribute to the development of new effective clinical strategies against mycoses. Nevertheless, the development of such a group of phosphors will allow us to identify new structures necessary for establishing a mechanism for regulating the molecular functioning of cells and a detailed study of cell morphology. This will provide new insights necessary for understanding the physiological adaptation of multicellular organisms and will also contribute to the development of new effective clinical strategies against mycoses.

**Figure 1:**
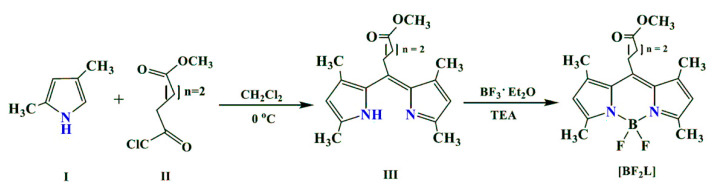
The synthesis scheme of [BF_2_L].

In order to create new intensely fluorescent, photostable and insensitive to the polarity of solvents biomarkers, we have synthesized a 3,3′,5,5′-tetramethyl-2,2′-dipyrromethenate boron(III), substituted in the *meso*-position by a (CH_2_)_3_COOCH_3_-radical (Figure 1). The data of X-ray structural analysis, the spectral-luminescent properties and processes of photochemical transformations in organic solvents of various nature, including those simulating biological media, have been studied in detail. Moreover, biochemical studies have been carried out for the first time in order to justify the potential possibilities for the practical use of the synthesized phosphor. In particular, it might be useful to examine cellular responses to a broad spectrum of agents, including antibiotics and chemotherapeutic drugs, and monitor the redistribution of the cellular organelles and reorganization of the membranes.

## 2. Results and Discussion

### 2.1. Synthesis of BODIPY

BF_2_-*meso*-(4-methoxycarbonylpropyl)-3,3’,5,5’-tetramethyl-2,2’-dipyrromethene was synthesized according to the procedure presented below. 

Dipyrromethene (**III**) was obtained by condensing 2,4-dimethylpyrrole (**I**) with dibasic acid monomethyl ester (**II**), then dye **III** was converted into the key product ([BF_2_L]) by the action of boron trifluoride etherate in the presence of triethylamine. A description of the synthesis procedure and the results of the analysis of synthesized BODIPY in the experimental part of the article are presented.

### 2.2. X-Ray Data

The compound [BF_2_L] is crystallized in the monoclinic space group *P*2_1_/*n* (Supplementary Figure S1). As previously observed in some similar structures [10,14,15,16], the BODIPY core B(N_2_F_2_) shows a quasi-tetrahedral configuration with a 108.8^0^ (12) F–B–F angle and a B–N and B–F average bond length of 1.5435 and 1.3958 Å, respectively (Supplementary Table S1, Figure S1). In the BODIPY core, the C_9_BN_2_ framework consisting of one central six-membered and two adjacent five-membered rings is nearly flat. This geometry indicates the strongly delocalized π-system nature of the C_9_BN_2_ framework in crystal, which is additionally confirmed by the bond lengths in this fragment. It was revealed that the introduction of the methyl groups at C9 and C10 positions in the BODIPY core imposes restrictions on the conformational mobility of the substituent in the *meso*-position of the BODIPY moiety (Supplementary Table S2).

So, in the [BF_2_L] molecule, orthogonal conformation has been observed in the first fragment of the alkyl substituent (the torsion angle C^2^C^3^C^17^C^18^ is close to 90 degrees), as well as in the transoid conformation of the second fragment (torsion angle C^3^C^17^C^18^C^19^ is close to 180 degrees). In fact, this may lead to end the steric influence of methyl groups. That is, the *gaushe*-conformation in the third link fragment (torsion angle C^17^C^18^C^19^C^20^ is close to 60 degrees). The conformation of the last C^18^C^19^C^20^O^22^ link is *gaushe*.

### 2.3. Spectroscopic Properties

**Figure 2:**
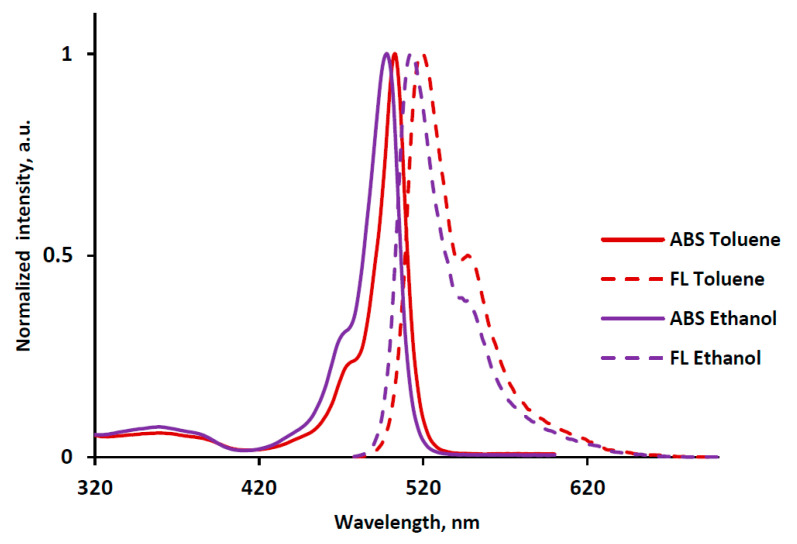
Normalized electronic absorption and emission spectra of [BF_2_L] solution in organic solvents.

The spectroscopic properties of the BODIPY were studied in a series of organic solvents: non-polar cyclohexane, aromatic toluene, electron-donating dimethyl sulfoxide and proton donor chloroform, ethanol, butanol-1 and octanol-1. The spectral characteristics of BF_2_-*meso*-(4-methoxycarbonylpropyl)-3,3’,5,5’-tetramethyl-2,2’-dipyrromethene are shown in Figure 2 and Table 1.

The electronic absorption spectrum of [BF_2_L] has a typical two-band form, in which the high-intensity *S*_0_ → *S*_1_ band has a maximum at the wavelength of 498–503 nm and a shoulder on the left slope at 471–476 nm, and the broadened less intense *S*_0_ → *S*_2_ band was observed in the shorter wavelength region at *λ*${}_{\max}^{\mathrm{abs}}$= 358–364 nm (Table 1, Figure 2). The values of the extinction coefficients (*ε*) of the first intense band are high enough and range from ~56,000 to 73,000 L·mol^−1^·cm^−1^ (Table 1) and are comparable with those for other *meso*-substituted BODIPYs [12,17,18]. The synthesized complex intensively fluoresces in the blue-green region of the spectrum. The BODIPY fluorescence spectra are mirror reflection of the corresponding absorption spectra (Figure 2). The maximum fluorescence band (*λ*${}_{\max}^{\mathrm{fl}}$) of the complex is observed at the wavelength range of 513–520 nm (at *λ*_ex_ = 470 nm, Figure 2, Table 1).

The influence of the medium properties was manifested in a slight narrowing of the intense absorption (fluorescence) bands and a hypsochrome shift of their maxima with increasing medium polarity. So, the electronic absorption spectra bands maxima were shifted hypsochrome to ~5 nm, and the fluorescence spectra to ~7 nm upon the transition from nonpolar and weakly polar (cyclohexane, toluene, chloroform) solvents to polar media (ethanol, butanol-1, octanol-1, DMSO) (Table 1). A larger hypsochromic shift (to ~8 nm) of the maxima of the absorption and fluorescence bands is observed for the meso-carboxyethyl- and meso-carboxypropyl-substituted analogs [18]. The observed small spectral changes can be explained by a slight increase in the dipole moment of the BODIPY molecules during the transition from the ground to the excited state. In the studied solvents, the Stokes shift showed the maximum value in aromatic toluene and electron-donating dimethyl sulfoxide (from 14 to 17 nm) (Table 1). The observed effect can be caused by the high solvating ability of dimethyl sulfoxide and the π-π-stacking of aromatic toluene molecules and pyrrole rings of the dipyrromethene core.

The introduction of a long alkyl substituent with an ester group into the *meso*-position of the indacene core leads to a noticeable increase in the Stokes shift as compared to Δ*ν_st_* = 200–355 cm^−1^ for the *meso*-unsubstituted BODIPY analogues [19]. The observed increase in the Stokes shift can be caused by structural differences in the ground and excited states of the [BF_2_L] luminophore due to the conformational chirality and mobility of the *meso*-substituent.

It was found that the fluorescence quantum yield of [BF_2_L] was maximum (~100%) in non-polar and weakly polar media (cyclohexane, toluene), while slightly reduced (to ~80–97%) in chloroform and alcohols (Table 1). An increase in the quantum yield of [BF_2_L] in the sequence of alcohols: ethanol, butanol-1 and octanol-1, could be caused by a decrease in the mobility of the *meso*-substituent with increasing viscosity of the medium. Therefore, a similar behavior of luminophore can be assumed in cell membranes and other lipid biostructures. Noticeable [BF_2_L] fluorescence quenching was observed in electron-donating DMSO (up to ~75%). This was probably caused by the high polarity and solvating ability of the medium, which led to an increase in the nonradiative energy losses of the solvated luminophore molecule in the excited state.

It should be noted that the introduction of methyl substituents in the 3,3’,5,5’-positions of the pyrrole rings noticeably enhances the fluorescence of the phosphor. For example, in ethanol, the value of the fluorescence quantum yield of BF_2_-*meso*-(4-methoxycarbonylpropyl)-3,3’,5,5’-tetramethyl-2,2’-dipyrromethene increases ~1.3 times compared to unsubstituted in the pyrrole rings BF_2_-*meso*-(4-carboxypropyl)-2,2’-dipyrromethene [18]. The observed effect is apparently due to the manifestation of the electron-donating effect of the methyl groups on the BODIPY skeleton.

The fluorescence lifetime (*τ*) and the radiative process constants (*k*_rad_) were found to be in the ranges of 12.0–16.0 ns and (5.96–8.33)·10^7^ s^–1^ respectively, and slightly depended on the medium properties (Table 1). The nature of the solvent had a more significant effect on the values of the non-radiative constants (*k*_nr_) of [BF_2_L] deactivation. For example, *k*_nr_ values increased by more than ~5–14 times in DMSO and ethanol in comparison with other studied media, thus indicating an increase of the solvation process of the luminophore. 

### 2.4. Partition Coefficient Determination

The lipophilicity of the compound [BF_2_L] was assessed by determination of the logP values between an aqueous phase and an octanol phase using the “shake-flask” method. The studied phosphor was majorly in the organic phase. To analyze the trend in the logP values, we compared the logP values for the synthesized [BF_2_L], BODIPY with *ms*-(4-methoxycarbonylbutyl) substituent and commercially available *meso*-unsubstituted analogue in the octanol-1/water model system [20]. Partition coefficients were 1.39, 1.68 and 1.80 for BF_2_-3,3′,5,5′-tetramethyl-2,2’-dipyrromethene, BF_2_-*ms*-(4-methoxycarbonylbutyl)- 3,3’,5,5’-tetramethyl-2,2’-dipyrromethene and BF_2_-*ms*-(4-methoxycarbonylpropyl)-3,3’,5,5’-tetramethyl-2,2’-dipyrromethene respectively, indicative of their high lipophilicity. This result was expected as the complexes are uncharged and bear lipophilic fragments. As expected, the introduction of a methoxycarbonylpropyl substituent into the BODIPY core *meso*-position is accompanied by luminophore hydrophobicity increasing (almost ~1.3 times), compared to the *meso*-unsubstituted analog. It should be noted that a decrease in the length of the *meso*-substituent by one -CH_2_-fragment somewhat increases the affinity of the phosphor [BF_2_L] for lipid structures in comparison with the ms- (4-methoxycarbonylbutyl-substituted) analog. This effect can be caused by intramolecular interaction in the case of a compound with a more extended *meso*-substituent, which, according to *X*-ray diffraction data, bends towards pyrrole rings [20]. These results indicate that synthesized [BF_2_L] can be used for labeling hydrophobic regions of blood plasma proteins and biological objects’ visualization.

### 2.5. Photostability

The stability of luminophores under the influence of UV irradiation is an important indicator for their practical use, including as fluorescent biomarkers. Our previous studies [21,22] showed that the photochemical degradation of open-chain oligopyrroles complexes occur due to redox processes involving oxygen, pyrrole and *meso*-substituents and heteroatoms. The nature of the medium had a significant effect on the efficiency of the photodegradation processes. The results of the analysis of the boundary molecular orbitals (LUMO and HOMO) energy levels [23,24] allowed to conclude that the methine *meso*-spacer and nitrogen atoms of the pyrrole rings are most photoactive in dipyrromethene dyes. An effective localization of electron density on these groups and atoms is observed in an excited state, which is conducive to the flow on them of redox reactions. As a result of this, BODIPY luminophores with an unsubstituted *meso*-spacer are primarily attacked by singlet oxygen against the methine group (=CH-) of the central spacer. This leads to discoloration of the dye as a result of redox processes, causing a violation of the π-conjugation of the pyrrole aromatic systems and the subsequent destruction of the indacene skeleton to unpainted pyrrole-containing products. Therefore, the study of the effect of dipyrromethene dyes’ substitution in the *meso*-spacer on their photostability is one of the actual tasks which requires to expand the practical application areas of this class of compounds.

**Figure 3:**
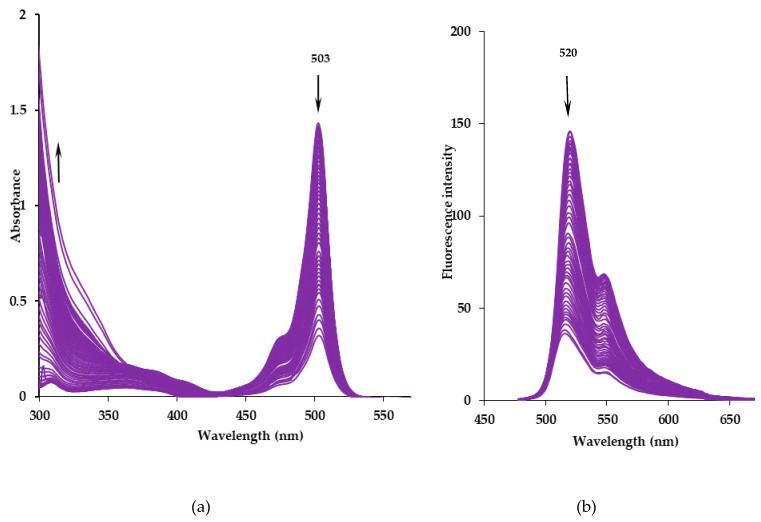
The changes of the electronic absorption (**a**) and fluorescence (**b**) spectra of a [BF_2_L] solution in toluene under UV irradiation.

The results of [BF_2_L] solutions photochemical destruction processes studies under the influence of UV radiation in nonpolar cyclohexane and aromatic toluene are presented in Table 2. Figure 3 shows a typical spectral picture of the electronic absorption spectra evolution of a [BF_2_L] solution in toluene under UV irradiation. 

The photodestruction of the [BF_2_L] solution upon UV irradiation in both solvents was accompanied by a decrease in the intensity of the electronic absorption spectrum characteristic bands in the range from 400 to 550 nm and an increase of absorption in the short-wave UV region (Figure 3a). The photodestruction process is also accompanied by a decrease in intensity and a small (up to 4 nm) blue shift of the maximum of the intense emission band (Figure 3b). Ultimately, complete photobleaching of the solutions was observed due to the destruction of the conjugated π-system of the chelate.

It was noted that photochemical degradation of [BF_2_L] under UV irradiation proceeds more efficiently in toluene than in cyclohexane (Table 2, Supplementary Figure S2). The calculated half-life (*t*_1/2_) of the dye in toluene was almost ~2.2-fold less than in cyclohexane. However, the value of the observed rate photobleaching constant (*k_obs_*) was ~1.9-fold higher in toluene compared to cyclohexane. In fact, the observed differences can be caused by an increase in the polarization of the dipyrromethene ligand aromatic system due to π–π stacking with solvent molecules and the interaction with the active radical products of toluene photolysis under UV irradiation [25,26].

An analysis of the obtained characteristics of the photodegradation processes of [BF_2_L] showed that replacing the hydrogen atom in the BODIPY methine spacer by an extended 4-methoxycarbonylpropyl substituent significantly increases the photostability of the synthesized luminophore compared to the *meso*-unsubstituted analog (Table 2) [22]. For example, the half-life (***t*_1/2_**) of the synthesized BODIPY increased by almost ~2.4 times in toluene and almost ~1.9 times in cyclohexane compared with the 3,3’,5,5’-tetramethylated boron(III) dipyrromethenate. The observed differences can be caused by the manifestation of the steric screening effect of the *meso*-spacer, as well as the redistribution of the electron density in the chromophore molecule due to the manifestation of the *meso*-substituent ester group electronic effect.

### 2.6. Fluorescent Staining of Pathogenic Microorganisms

The possible use of [BF_2_L] as a fluorescent marker on pathogenic microorganisms has been analyzed by staining several strains of microscopic fungi C. albicans and F. Oxysporum with [BF_2_L] phosphor and then analyzing them by means of CLSM.

**Figure 4:**
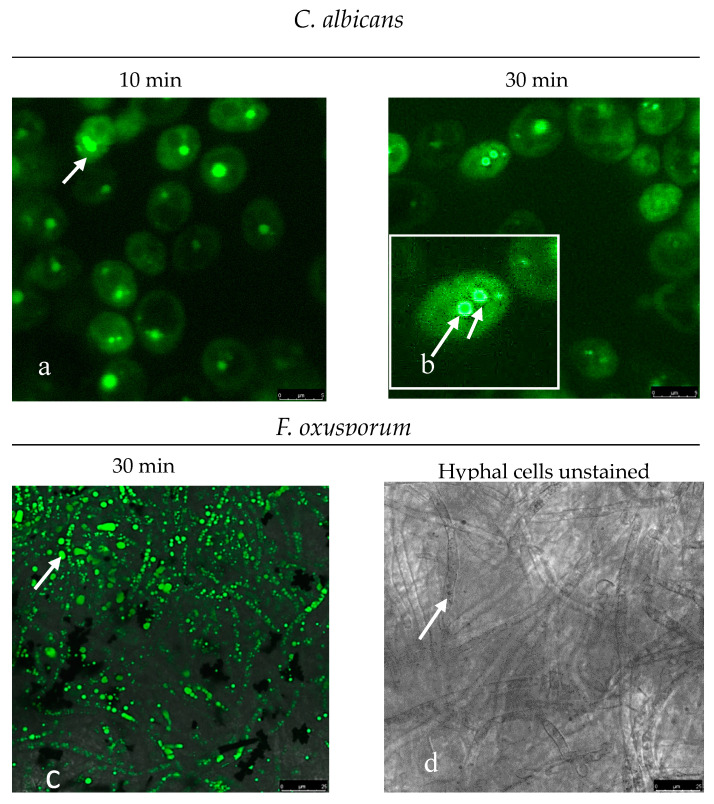
CLSM of fungal cells of C. albicans and F. oxysporum. The scale bar is 5 microns. Photos (**a**,**b**)—cells of C. albicans fungi (after 10–30 min staining with [BF_2_L], intensely stained nuclei and membranes of the structural components of the cell are visible. Photo (**c**)—hyphal, multinucleated fungus F. Oxysporum cells (after 30 min staining with [BF_2_L] intensely stained nuclei and membranes of the structural components of the cell are visible). Photo (**d**)—hyphae of the fungus F. Oxysporum (unpainted preparation).

It has been established that [BF_2_L] penetrates the cytoplasm of fungal cells after 10 min and stains the membrane structures of organelles where nuclei membranes are the most intensively stained. Actually, intense fluorescence was observed in the cells of the hyphal fungi F. Oxysporum, which contains several nuclei simultaneously. However, cell membrane staining was absent both in the yeast cell as well as in the hyphal one (Figure 4). 

In fact, this is consistent with the available literature data, which indicate that there is a difference in the molecular structure between the membrane of structural organelles and the cell membrane, where there are no sterol-rich domains [27].

It would seem that our findings allow us to use the synthesized [BF_2_L] phosphor as a marker in assessing the membrane structures of fungal cells, which may help in the study of the existing significant differences among a large number of mycotic infections pathogens. Moreover, it has been established that using [BF_2_L] is possible in determining effectors and modulating in vitro metabolic pathways of the fungal cell.

Interestingly, after Dox treatment, the [BF_2_L]-mediated fluorescence pattern was redistributed from diffuse to the micro-speckled one. Moreover, the staining pattern was detected as predominantly membranous, thereby illustrating the reorganization in the cellular membranes in the cells that were exposed to chemotherapeutic agent.

### 2.7. Fluorescent Staining of Mammalian Cells

**Figure 5:**
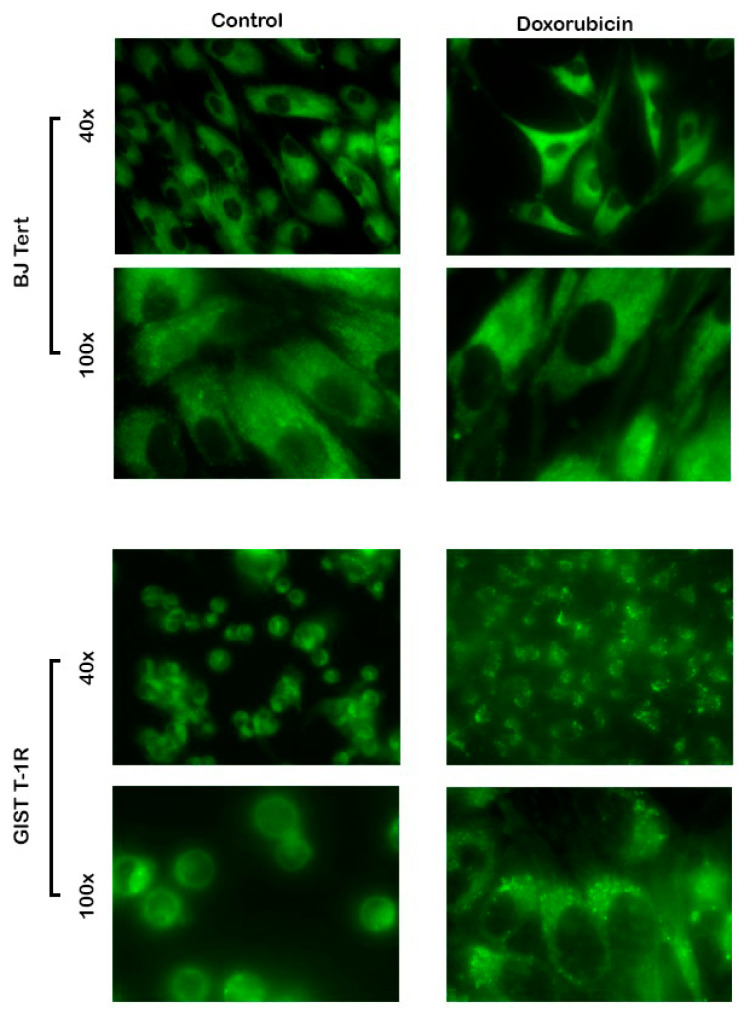
Changes in [BF_2_L]-mediated fluorescence as a potential marker of reorganization of Table 1. R cancer cells (lower panel) pretreated with doxorubicin (0.5 µg/mL) for 24 h or left untreated (control) prior to the [BF_2_L] exposure (1 h). The experiments were performed in triplicates. The images were shown at 40x and 100x magnifications for each experimental condition.

No differences in [BF_2_L]-mediated fluorescence were observed between non-treated BJ tert fibroblasts and gastrointestinal stromal tumors (GIST) cells (Figure 5—left panel). This was found for cellular staining pattern and fluorescence intensity, as well. However, an increased intensity of [BF_2_L]-induced fluorescence was detected after Dox treatment in both transformed and non-transformed human cell lines (Figure 5—right panel). Interestingly, after Dox treatment, the [BF_2_L]-mediated fluorescent pattern was redistributed from diffuse to the micro-speckled one. Moreover, the staining pattern was detected as predominantly membranous, thereby illustrating the reorganization in the cellular membranes in the cells that were exposed to chemotherapeutic agent.

Given that Dox is a well-known chemotherapeutic agent exhibiting potent proapoptotic activity, the changes in fluorescence pattern in Dox-treated cells might reflect the structural changes in the plasma membranes that are necessary for the clearance of apoptotic cells. In particular, these changes might reflect the expression of phosphatidylserine (PS) on the outer side of the membrane and budding of the membrane into microvesicles and apoptotic bodies. Further studies are needed to reveal this proposal.

**Figure 6:**
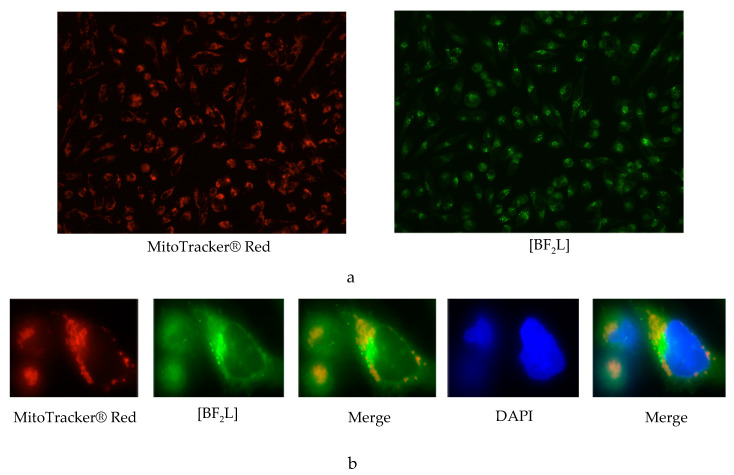
Subcellular distribution of [BF_2_L] (green) co-stained with MitoTracker® Red CMXRos (red) in GIST T-1R cancer cells. Cells were stained with DAPI (blue) to outline the nucleus. The experiments were performed in the triplicates. The images were shown at 20x (**a**) and 100x (**b**) magnifications.

We also examined a subcellular localization of [BF_2_L] and its possibility to be selectively accumulated in the cellular organelles, such as mitochondria. For this purpose, we stained mammalian cells with MitoTracker® Red CMXRos together with [BF_2_L] for 45 min and proceeded the cell slides for immunostaining. We found a weak co-localization pattern between these dyes, thereby suggesting that [BF_2_L] is not selectively accumulated in the mitochondria of mammalian cells (Figure 6). Further studies are also needed to examine whether the other cellular organelles might selectively accumulate [BF_2_L].

## 3. Experimental 

### 3.1. Chemistry

The cyclohexane, toluene, chloroform, ethanol, butanol-1, octanol-1 and DMSO (Panreac, Barcelona, Spain) in the present study have been used without further purification. 

Synthesis of BF_2_-*meso*-(4-methoxycarbonylpropyl)-3,3’,5,5’-tetramethyl-2,2’-dipyrromethene (BF_2_C_18_H_23_N_2_O_2_; *M* = 348.2) was performed under argon with stirring on a magnetic stirrer according to the procedure presented in Reference [11]. A 0.97 mL solution (0.8990 g, 9.45 mmol) of 2,4-dimethylpyrrole was added slowly drop by drop to a 0.65 mL solution (0.7781 g, 4.73 mmol) of methyl 4-chloroformyl butyrate in 40 mL of dried and cooled (to 0 °C) methylene chloride. Next, the obtained mixture was maintained at room temperature. After that, a 2 mL of triethylamine was added to the solution at room temperature and stirred on a magnetic stirrer, followed by adding 1.5 mL of BF_3_·Et_2_O (~12 mmol). The solution was set to stir at room temperature for 3 h. In fact, the complex formation was monitored by changes of ABS by the disappearance of the absorption band of the ligand in the electronic spectrum. At the end, the solvent was evaporated at low pressure, and the solid residue was dissolved and chromatographed on silica gel, eluting by 1:1 petroleum ether:methylene chloride. The eluate was evaporated, and the product was precipitated with methanol from a concentrated solution in dichloromethane under cooling. The obtained yield was 0.2431 g (0.697 mmol, 14.7%). ^1^H NMR spectrum (CDCl_3_), δ, ppm: 6.08s (2H, 4,4-H); 3.72s (3H, OCH_3_); 2.99–3.05 m (2H, *ms*-CH_2_); 2.53 s + 2.52 t (6 + 2 H, *J* = 7.2 Hz, CH_3_ + CH_2_CO); 2.45 s (6 H, CH_3_); 1.94–2.02 m (2 H, CH_2_) (Supplementary Figure S3). Mass spectrum, *m/z*: 329.50 [*M*-F]^+^ (Supplementary Figure S4). Found, %: H 6.69, C 62.01, N 8.13. BF_2_C_18_H_23_N_2_O_2_. Calculated, %: H 6.66, C 62.09, N 8.05. 

### 3.2. NMR Studes

The NMR experiment was performed on a Bruker Avance III 500 MHz NMR spectrometer equipped with a 5 mm probe using standard Bruker TOPSPIN software. Experiments were carried out without sample spinning. ^1^H-NMR (500.17 MHz, CDCl_3_, 25 °C) chemical shifts were referenced to residual solvent signal CDCl_3_. 

### 3.3. MALDI TOF Studes

Mass spectrum was recorded on a Shimadzu AXIMA Confidence MALDI TOF-TOF mass spectrometer in positive ion reflectron mode.

### 3.4. Elemental Analyses

Elemental analyses (C, H, and N) were carried out on a Flash EA 1112 Elemental analyzer. 

### 3.5. UV-Vis Spectroscopy 

Electronic absorption and fluorescence spectra of **[BF_2_L]** solutions were recorded on a CM 2203 spectrofluorimeter (SOLAR) in the range of 10^−7^ to 10^−5^ mol·L^−1^ molar concentrations and 10 mm of absorbing layer thickness at *t* = 25 ± 0.1 °C. Fluorescence spectra were obtained at an optical density less than 0.1 at the excitation wavelength. The fluorescence quantum yields (*φ*) of BODIPY were measured using the standard method (using Rhodamine 6G as standard). The error in determining the quantum yields luminescence is 10%.

The fluorescence lifetime (*τ*) was estimated based on the spectral-luminescent characteristics. In accordance with *φ* = *k*_rad_/(*k*_rad_ + *k*_nr_) and *τ* = 1/(*k*_rad_ + *k*_nr_), fluorescence lifetime was determined by: *τ* = *φ*/*k*_rad_, where *k*_nr_ is the rate of non-radiative processes, *k*_rad_ is the rate of radiative processes (radiation constant) and the fluorescence lifetime error is ~10–15%. The rate constant of radiative processes was estimated from the characteristics of the electronic absorption spectra in accordance with Reference [28]: *k*_rad_ = 2.9 × 10^–9^ × [(9n_D_^2^)/(n_D_^2^ + 2)^2^ ] × *ν*_max_^2^ × *ε*_max_ × Δ*ν*_1/2_, where *n*_D_ is the refractive index of the solvent, *ν* is the wave number of the absorption band maximum (cm^−1^), Δ*ν*_1/2_ is half-width of the absorption band (cm^–1^) and *ε* is extinction coefficient of intense absorption band. The rate constant of non-radiative deactivation (*k*_nr_) was calculated from experimentally measured quantum yield and lifetime according to the following equation: *k*_nr_ = (1 – *φ*)/*τ*.

Partition coefficient determination: The lipophilicity of a compound was determined by measuring its distribution coefficient between the water and octanol phase by using the “shake-flask” method. The BODIPY (4.08–6.25 μM) was dissolved in octanol-1. A solution of complex in octanol-1 was mixed with water (1:1) with shaking for 8 h at room temperature. After equilibration, the octanol-1/water system was separated. The concentration of BODIPY in octanol-1 and water was determined using UV-Vis spectroscopy. The logP was calculated by the following equation:

logP = log([BODIPY]_octanol-1_/[BODIPY]_water_).

The photobleaching was carried out in quartz cells where sample solutions of BODIPY in cyclohexane or toluene (*c* = ~1·10^–5^ mol·L^−1^) were irradiated with a monochromatic light (mercury lamp 250 W with a light filter Carl Zeiss JENA, *λ* = 365 nm) at room temperature. The area of the light flux was 2.02 cm^2^ at a specific power W365 = 1.47 of mW/cm^2^ of a UV lamp. The irreversible bleaching of the dyes at the absorption peak was monitored as a function of time. Electronic absorption and fluorescence spectra of BODIPYs solutions were recorded at equal intervals of irradiation (through 30 min) on a spectrofluorometer SM2203 SOLAR in the 350–700 nm wavelength range. The half-life of the complex was defined as the time during which the chromophore is destroyed by 50%. The observed photooxidation rate constants (*k*_obs_) were determined by the equation ln(*A*_t_/*A*_0_) = *k*_obs_·*t*, where *A*_0_ and *A*_t_ are the initial and current optical density at the maximum of the long-wavelength absorption band respectively, and *t* is the time of irradiation of the sample with UV light. A similar approach to the calculation was applied by the authors of Reference [29].

### 3.6. X-ray Structure Determinations of [BF_2_L]

X-ray: A dataset for single crystals [BF_2_L] was collected on a Rigaku XtaLab Synergy S instrument with a HyPix detector and a PhotonJet microfocus *X*-ray tube using Cu Kα (1.54184 Å) radiation at 100 K. Images were indexed and integrated using the CrysAlisPro data reduction package. Data were corrected for systematic errors and absorption using the ABSPACK module. The GRAL module was used for analysis of systematic absences and space group determination. Using Olex2 [30], structure was solved by direct methods with SHELXT [31] and refined by the full-matrix least-squares on F^2^ using SHELXL [32]. Non-hydrogen atoms were refined anisotropically. The figures were generated using the Mercury 4.1 [33] program, CCDC number 2011215. 

Crystal data for C_18_H_23_BF_2_N_2_O_2_ (*M* = 348.19 g/mol): monoclinic, space group *P*2_1_/*n* (no. 14), *a* = 8.7003(2) Å, *b* = 13.5758(3) Å, *c* = 14.4947(3) Å, *β* = 94.757(2), *V* = 1706.12(7) Å^3^, *Z* = 4, *T* = 100.00(10) K, μ(CuKα) = 0.850 mm^−1^, *Dcalc* = 1.356 g/cm^3^, 21,076 reflections measured (8.94° ≤ 2 Θ ≤ 154.29), 3525 unique (*R*_int_ = 0.0772, R_sigma_ = 0.0430) which were used in all calculations. The final *R*_1_ was 0.0512 (I > 2σ(I)) and *wR*_2_ was 0.1443 (all data). Crystal structure assay was supported by the Government assignment for the FRC Kazan Scientific Center, Russian Academy of Sciences.

### 3.7. Biological Studies

Biological assays—strains and growth conditions: Candida albicans C5050-19 yeast (clinical isolate isolated from the mucous pharynx) and Fusarium oxysporum C2413-19 mycelial fungus (clinical isolate isolated from the skin) were used in this study. Colonies were grown on Saburo medium at 30 °C for 2–5 days, respectively. A working suspension was prepared by adjusting the optical density of 0.5 according to McFarland in a 0.9% NaCl solution.

Oncological assays: Doxorubicin (Dox) was purchased from Sigma-Aldrich, St. Louis, USA. For cell lines and culture conditions, Human IM-resistant GIST T-1 cell line (T-1R) used in the present study was established in our laboratory from parental GIST T- cell line [34] after a continuous induction from 0.4 to 1000 nM IM in a stepwise increasing concentration manner [35]. GIST T-1R cells were maintained in RPMI-1640 medium supplemented with 10% fetal bovine serum (FBS) (HyClone, USA), 1% L-glutamine, 50 U/mL penicillin and 50 µg/mL streptomycin (Paneko, Moscow, Russia). BJ tert human fibroblasts (kindly provided by O. Gjoerup, University of Pittsburgh, USA) were grown in 80% Dulbecco’s modified Eagle’s medium and 20% medium 199 (both from Paneko), supplemented with FBS and antibiotics, as indicated above. The cells were cultured in a humidified atmosphere of 5% CO_2_ at 37 °C (LamSystems, Miass, Russia).

### 3.8. Confocal Laser Scanning Microscopy

Fungal cells were stained using **[BF_2_L]** (2 mg/mL) for 10–30 min. Stained cells were analyzed in vivo using an LSM780 reverse confocal laser scanning microscope (Germany, Jena, Carl Zeiss AG) along a green (488/490–606 nm) channel [36] (Sharafutdinov et al. IJMS, 2019). Microscopy data were processed using ZEN 9.0 software (Carl Zeiss AG).

### 3.9. Fluorescence Microscopy

Cells were seeded on glass coverslips coated with poly-L-lysine (St. Louis, MO, USA) and allowed to attach for 48 h before treatment. The cells were treated with Dox (0.5 μg/mL) for 24 h or left non-treated (control). Next, [BF_2_L] (20 μg/mL) was introduced into the cell culture for 1 h. After that, the cells were washed with phosphate-based saline (PBS) and the coverslips were mounted on glass slides. To examine the subcellular (i.e., mitochondria) localization of [BF_2_L], we used MitoTracker® Red CMXRos (Thermo Fisher Scientific, Waltham, MA, USA). This dye was introduced into cell culture (100 nM) and incubated for 45 min at 37 °C. After incubation, the cells were fixed in ice-cold methanol for 15 min at −20 °C, rinsed 3 times with PBS for 5 min and proceeded for immunostaining. The cells were visualized on an Olympus BX63 fluorescence microscope and the images were captured using a Spot advanced imaging system.

## 4. Conclusions

To sum up, a *meso*-(4-methoxycarbonylpropyl)-3,3’,5,5’-tetramethyl-2,2’-dipyrromethenate boron(III) behaves as a highly photostable fluorescent marker, emitting in the blue-green region (513–520 nm) with a high quantum yield (almost ~75–100%). It was found that fluorophore fluorescence exhibits maximum quantum yield (~100%) in non-polar and aromatic media (cyclohexane, toluene). However, this value decreases slightly (up to ~80–90%) in chloroform and alcohols. Moreover, it has been shown that increasing of [BF_2_L] fluorescence quantum yield in alcohols sequence, like ethanol, butanol-1 and octanol-1, is obviously caused by a decrease in the mobility of the extended *meso*-substituent with increasing viscosity of the medium. It has also been established that marked quenching of [BF_2_L] fluorescence is observed in electron-donating DMSO (up to ~75%). The observed effect may be due to the high polarity of the solvent. 

Moreover, it has been found that the photostability of the synthesized luminophore [BF_2_L] is ~2 times higher than that of the *meso*-unsubstituted analog, which can be caused by the steric effect of *meso*-position screening of dipyrromethenate boron(III) with a *meso*-(CH_2_)_3_COO-CH_3_-substituent, as well as electronic effects of the replacement of functional groups.

The evidence from this study suggests that using [BF_2_L] as a fluorescent marker in assessing the membrane structures of fungal cells may be useful in studying the significant differences among a large number of pathogens of mycotic infections. In addition to this, using [BF_2_L] is possible in determining effectors and modulating in vitro metabolic pathways of the fungal cell, as well as for visualizing changes in the structure in the plasma membrane, which is necessary for the clearance of apoptotic cells.

## Figures and Tables

**Table 1:** The spectroscopic characteristics of [BF_2_L] in organic solvents ^1^.

Solvent	*λ*${}_{\max}^{\mathrm{abs}}$, nm (*ε*, L·mol^−1^·cm^−1^) *S*_0_ → *S*_1_ *S*_0_ → *S*_2_	*λ*${}_{\max}^{\mathrm{fl}}$, nm (*λ*_ex_ = 470 nm) *S*_1_→*S*_0_	Δ*ν*_st_, cm^−1^	*φ* (*λ*_ex_ = 470 nm) *S*_1_ → *S*_0_	*k*_rad_·10^−7^, s^−1^	*k_n_*_r_·10^−7^, s^−1^	*τ*, ns
**cyclohexane**	502 (72,772) 476(sh) 358–361	516	541	1.000	8.33	0	12.0
**toluene**	503(70,323) 472(sh) 359–361	520	650	0.979	6.83	0.14	14.3
**chloroform**	502(66,317) 472(sh) 359–362	517	578	0.972	6.84	0.20	14.2
**octanol-1**	502(61,467) 472(sh) 358–364	516	541	0.969	6.06	0.19	16.0
**butanol-1**	499(59,849) 472(sh) 358–360	514	585	0.949	6.30	0.34	15.1
**ethanol**	498(60,491) 472(sh) 358–360	513	587	0.799	6.39	1.61	12.5
**DMSO**	498(56,469) 471(sh) 359–362	515	663	0.749	5.96	2.02	12.6

^1^*λ*${}_{\max}^{\mathrm{abs}}$, *λ*_ex_, *λ*${}_{\max}^{\mathrm{fl}}$— absorption, excitation and fluorescence maxima respectively, nm; sh—shoulder; *ε*—molar absorption coefficient (L·mol^−1^·cm^−1^); Δ*ν*_st—_Stokes shift, cm^−1^; *φ—*fluorescence quantum yield; *k*_rad_, *k_n_*_r_—rate constants of radiative process and nonradiative deactivation respectively, s^–1^; *τ—*fluorescence lifetime, ns.

**Table 2:** Quantitative characteristics of BODIPYs photostability in organic solvents.

Complexes	Solvent	*t*_1/2_, h	*k_obs_ ·*10^−6^, *s*^−1^
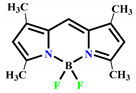 Difluoroborate 3,3′,5,5′-tetramethyl-2,2′-dipyrrometene [22]	cyclohexane toluene	46.0 16.7	3.3 ± 0.1 8.6 ± 0.6
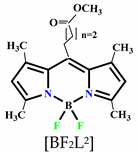	cyclohexane toluene	88.6 40.5	2.9 ± 0.2 5.5 ± 0.3

## Supplementary Materials

The following are available online, Figure S1: Geometry of the molecule [BF_2_L] in the crystal by X-ray data, Figure S2: Photobleaching curves of [BF_2_L] measured in toluene and cyclohexane solutions recorded at regular intervals following photo irradiation at 365 nm, Figure Figure S3: ^1^H-NMR spectrum of the [BF_2_L], Figure S4: Mass spectrum of the [BF_2_L]. Table S1: The main geometric parameters of BODIPY moiety for molecules of [BF_2_L] in a crystal according to *X*-ray diffraction data, Table S2: Selected torsion angle values for BODIPY [BF_2_L] molecule in a crystal according to X-ray diffraction data.
