# Supplementary material for: Spectroscopic and In Vitro Investigations of Boron(III) Complex with Meso-4-Methoxycarbonylpropylsubstituted Dipyrromethene for Fluorescence Bioimaging Applications
Source: Molecules. 2020 Oct 3;25(19):4541. doi: 10.3390/molecules25194541 (PMC7582871; doi:10.3390/molecules25194541)
Supplement: Supplementary file 1 [file molecules-25-04541-s001.pdf]

Supplementary data

Table S1. The main geometric parameters of BODIPY moiety for molecules of [BF_2L] in a crystal according to *X*-ray diffraction data.

**Bond**	**Distance, Å**
N^1-C^7	1.3488(19)
C^7-C^8	1.398(2)
C^8-C^9	1.383(2)
C^9-C^2	1.431(2)
C^2-C^3	1.403(2)
C^3-C^4	1.408(2)
C^4-C^{10}	1.427(2)
C^{10}-C^{11}	1.387(2)
C^{11}-C^{12}	1.393(2)
C^{12}-N^5	1.3510(18)
C^4-N^5	1.4056(18)
N^1-B^6	1.5459(19)
N^5-B^6	1.541(2)
B^6-F^6	1.3929(18)
B^6-F^7	1.3987(17)

Table S2. Selected torsion angle values for BODIPY [BF_2L] molecule in a crystal according to *X*-ray diffraction data.

**Angles, degrees**	
$C^2C^3C^{17}C^{18}$	86.23(16)
$C^3C^{17}C^{18}C^{19}$	-166.06(12)
$C^{17}C^{18}C^{19}C^{20}$	-53.68(17)
$C^{18}C^{19}C^{20}O^{22}$	-51.05(17)

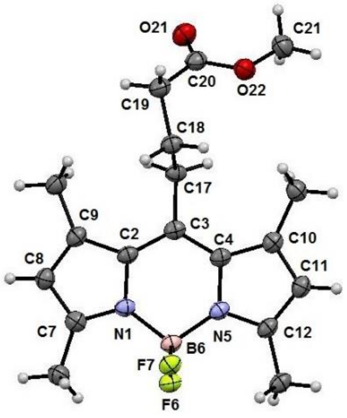

Figure S1. Geometry of the molecule [BF_2L] in the crystal by *X*-ray data.

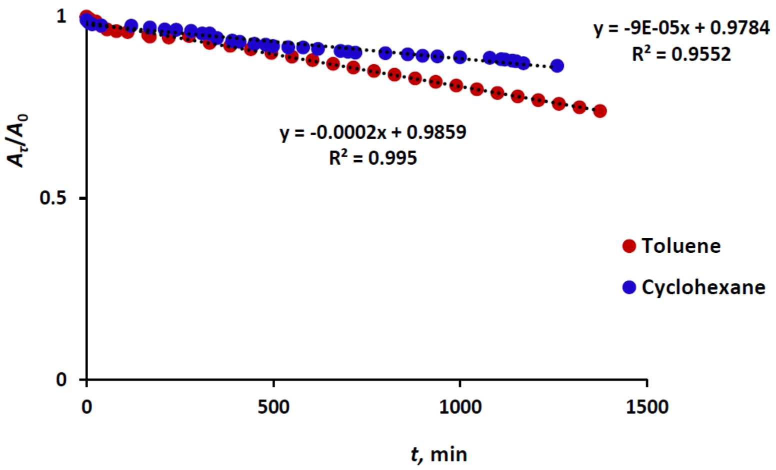

Figure S2. Photobleaching curves of [BF_2L] measured in toluene and cyclohexane solutions recorded at regular intervals following photo irradiation at 365 nm.

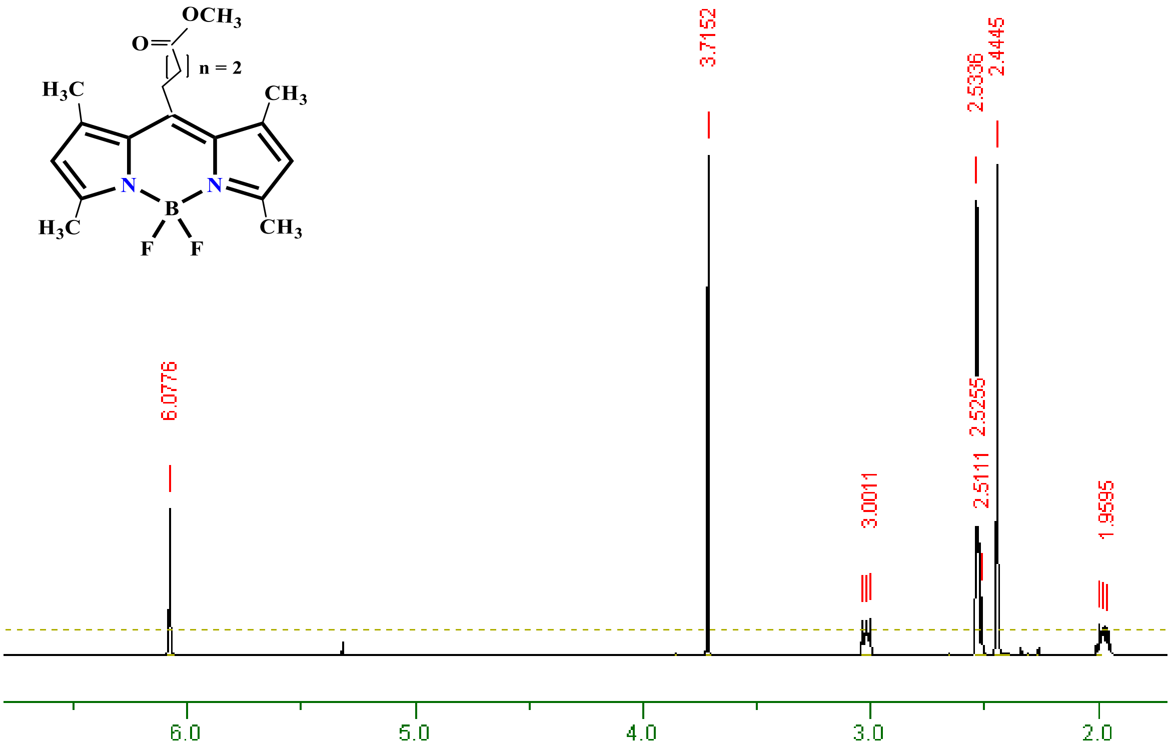

^{1}H NMR spectrum ($CDCl_3$), δ, ppm: 6.08s (2H, 4,4'-H); 3.72s (3H, OCH_3); 2.99-3.05m (2H, *ms*-CH_2); 2.53s+2.52t (6+2H, J = 7,2Hz, CH_3+CH_2CO); 2.45s (6H, CH_3); 1.94-2.02m (2H, CH_2).

Figure S3. ^{1}H NMR spectrum of the [BF_2L].

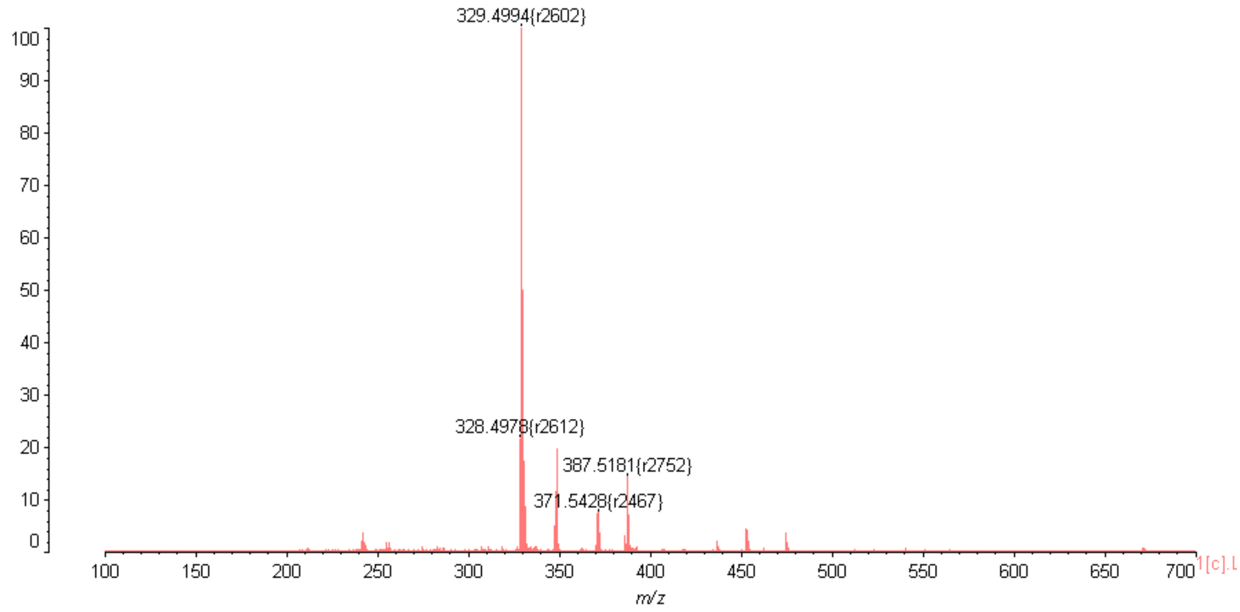

Figure S4. Mass spectrum of the [BF_2L].
